# A cascade of protein aggregation bombards mitochondria for neurodegeneration and apoptosis under WWOX deficiency

**DOI:** 10.1038/cddis.2015.251

**Published:** 2015-09-10

**Authors:** C I Sze, Y M Kuo, L J Hsu, T F Fu, M F Chiang, J Y Chang, N S Chang

**Affiliations:** 1 Department of Cell Biology and Anatomy, National Cheng Kung University, Tainan, Taiwan, ROC; 2 Department of Medical Laboratory Science and Biotechnology, National Cheng Kung University, Tainan, Taiwan, ROC; 3 Department of Neurosurgery, MacKay Memorial Hospital Taipei, and Graduate Institute of Injury Prevention and Control, Taipei Medical University, Taipei, Taiwan, ROC; 4 Institute of Molecular Medicine, College of Medicine, National Cheng Kung University, Tainan, Taiwan, ROC; 5 Advanced Optoelectronic Technology Center, National Cheng Kung University, Tainan, Taiwan, ROC; 6 Department of Neurochemistry, New York State Institute for Basic Research in Developmental Disabilities, Staten Island, NY, USA

The presence of protein aggregates or inclusion bodies in the neurons or glial cells is one of the pathological hallmarks of neurodegenerative diseases, such as tauopathy in Alzheimer's diseases (AD). Infectious agents or unknown etiological factors may cause *de novo* misfolding of peptides or proteins to form proteinaceous seeds or oligomers. These protein seeds lead to self-propagation of pathologic aggregates that impair neuronal functions at different brain regions for causing specific neurological diseases.^1^

Substantial evidence reveals that tumor suppressor WW domain-containing oxidoreductase, designated WWOX, FOR or WOX1, controls protein aggregation in the human brain.^2^ Downregulation of WWOX appears to start in the middle ages that leads to the slow progression of neurodegeneration.^2^ WWOX is a multi-functional molecule involved in cell signaling, cancer progression, metabolic disorders, and neural diseases.^2^ When WWOX protein is totally lost due to alteration of *WWOX*/*Wwox* gene such as missense or nonsense mutation and deletion, neural disorders, and metabolic diseases occur *in vivo*, including ataxia, epilepsy, dementia, neurodegeneration, growth retardation, abnormal HDL lipid metabolism, and early death.^2, 3^

WWOX blocks neurodegeneration by binding tau and tau-hyperphosphorylating enzymes GSK3*β*, ERK, and JNK1, (refs 4, 5, 6) and promotes neuronal differentiation.^5^ WWOX interacts with ERK and JNK1 via its N-terminal first WW domain, but binds tau and GSK3*β* via C-terminal short-chain alcohol dehydrogenase/reductase (SDR) domain.^4, 5^ A portion of WWOX is in the mitochondria, so as to maintain the normal cell physiology. The SDR domain appears to have a key role in the mitochondrial homeostasis.^4, 5, 6^ WWOX is frequently downregulated in the AD hippocampi.^2, 4^ This downregulation results in spontaneous relocation of TGF*β*1-induced anti-apoptotic factor 1 (TIAF1) and TRAPPC6AΔ (trafficking protein particle complex 6A delta, TPC6AΔ) to the mitochondria and both proteins become aggregated (see Figure 1).^6, 7^ These aggregated proteins activate caspases, which leads to tau tangle formation, amyloid precursor protein (APP) degradation, formation of amyloid beta (A*β*), and plaques in humans and in *Wwox* gene knockout mice.^5, 6, 7^

In an inaugural issue of *Cell Death Discovery*, Chang *et al.*^8^ described a protein aggregation cascade, involving TRAPPC6AΔ, TIAF1, A*β* and tau, under the influence of transforming growth factor beta (TGF-*β*) or WWOX deficiency. In physiologic settings, TGF-*β*1 induces endogenous wild-type TPC6A and TPC6AΔ to undergo shuttling—back and forth between nucleoli and mitochondria in 40–60 min in *Wwox* knockout MEF cells (see Figure 1a). WWOX reduces the shuttling time by ~50% in wild-type cells.^8^ Both TPC6A and TPC6AΔ bind to the C-terminal tail of WWOX. Unlike the wild type, TPC6AΔ readily forms aggregates or plaques intracellularly or extracellularly in the brain cortex and hippocampus.^7^ Formation of TPC6AΔ aggregates precedes A*β* generation in the hippocampi of middle-aged postmortem normal humans.^7^ The plaques of pT181-Tau and TPC6AΔ are found in the cortex and hippocampus in 3-week-old *Wwox* gene knockout mice, indicating a significant increase in protein aggregation under WWOX deficiency.^7^ The knockout mice survive less than 1 month.

It appears that under aberrant signaling, protein aggregation occurs. By time-lapse microscopy, TGF-*β*1 initially increases the binding of ectopic TPC6AΔ with ectopic WWOX to a maximal extent in 4.5–5 h, followed by dissociation.^8^ TPC6AΔ undergoes Ser35 phosphorylation-dependent polymerization and binds TIAF1 for depositing onto the mitochondrial surface as aggregates (see Figure 1b).^6, 7, 8^ TIAF1 also undergoes Ser37 phosphorylation and then polymerizes. Caspase 3 becomes activated, and APP is Thr688-dephosphorylated and degraded to generate APP intracellular domain, A*β* and amyloid fibrils.^6^ Polymerized TIAF1 binds amyloid fibrils, which supports plaque formation *in vivo*.^6^ Smad4 of the TGF-*β* pathway blocks TIAF1 aggregation.^8^ When WWOX is knocked down by siRNA, aggregation of TPC6AΔ and TIAF1 occurs in the mitochondria to induce apoptosis.^6, 7, 8^ The N-terminal WW domain does not bind TPC6AΔ. When TGF-*β*1 induces Tyr33 phosphorylation in WWOX, this causes unfolding and exposure of WW domain for binding TPC6AΔ in the nucleus and thereby prevents aggregation and apoptosis.^8^ TGF-*β*1 probably uses membrane hyaluronidase Hyal-2 to signal the complex formation of Hyal-2 with WWOX and Smad4 to control SMAD-responsive promoter activation and protein aggregation.^9^ TGF-*β*1-mediated aggregation of TIAF1 can be independent of the TGF-*β* canonical signaling.^6^

Together, WWOX is required for the survival of organisms. It regulates many pathophysiological processes for blocking neurodegeneration, and functions as a multi-tasked molecule among protein interaction networks. Restoration of WWOX is expected to help survival of neural cells by preventing accumulation of protein aggregates in neurons. A small Tyr33-phosphorylated WWOX peptide,^10^ which mitigates MPP+-mediated Parkinson-like syndrome in rats, may be of therapeutic use in the restoration of neural function under WWOX deficiency *in vivo*.

## Figures and Tables

**Figure 1:**
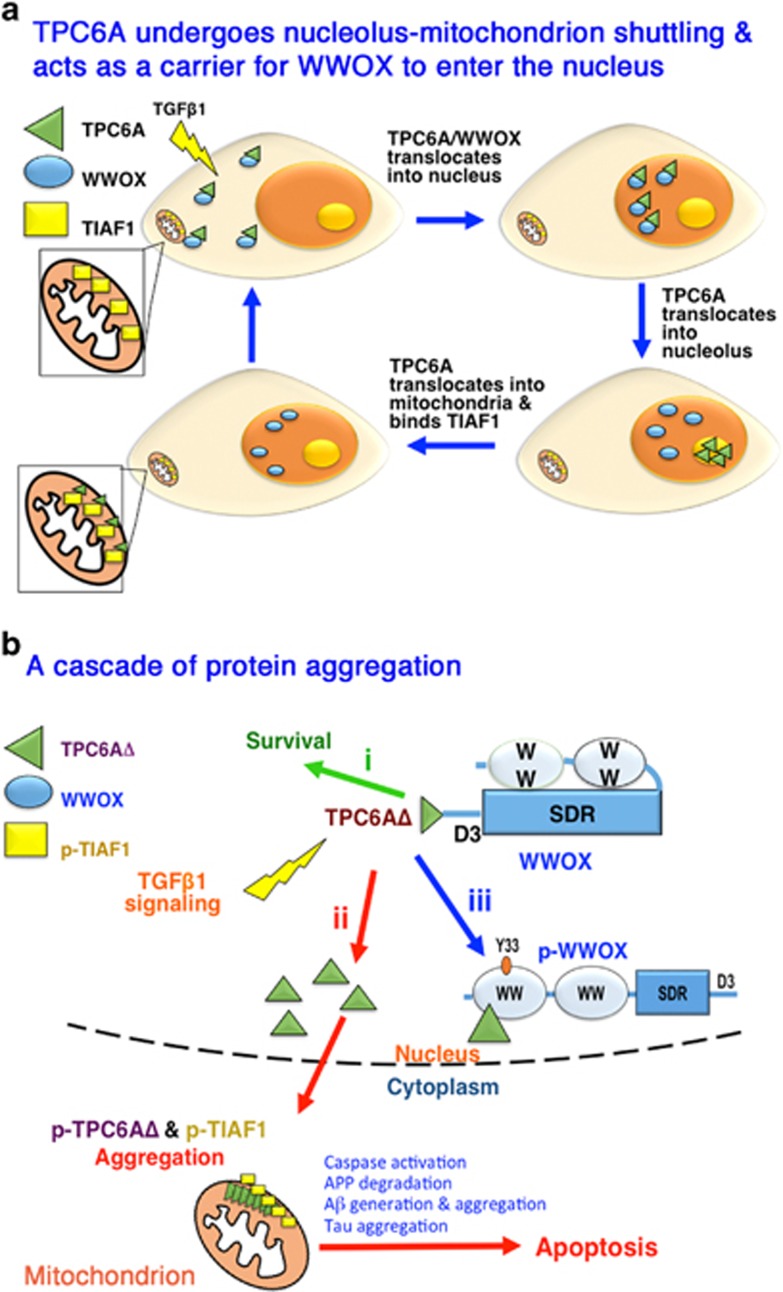
A cascade of protein aggregation bombards mitochondria. (**a**) WWOX physically interacts with wild-type TPC6A and TPC6AΔ in the cytoplasm. TGF-*β*1 induces nucleolus–mitochondrion shuttling of TPC6A and TPC6AΔ.^8^ TPC6A or TPC6AΔ acts as a carrier to enhance WWOX nuclear accumulation. WWOX continues to stay in the nucleus, whereas TPC6A relocates to the nucleolus. Later, TPC6A relocates to the mitochondria, where it binds Ser37-phosphorylated TIAF1.^8^ Ser35 phosphorylation is needed for TPC6A to relocate to the nucleolus, and Tyr112 phosphorylation needed for cytoplasmic translocation. (**b**) In the route **i** (green), the C-terminal tail of WWOX binds and inhibits TPC6AΔ polymerization. No cell death occurs. In the route **ii** (red), under aberrant TGF-*β* signaling, both TIAF1 and TPC6AΔ are accumulated on the surface of the mitochondria and become aggregated for causing caspase activation, APP degradation, A*β* generation, tau aggregation, and/or apoptosis.^6, 7, 8^ In the route **iii** (blue), activated WWOX with Tyr33 phosphorylation in the nucleus allows binding of TPC6AΔ to its first WW domain, and thereby prevents TPC6AΔ aggregation
